# PYCR1在肿瘤发生发展中的作用和机制研究进展

**DOI:** 10.3779/j.issn.1009-3419.2026.106.04

**Published:** 2026-02-20

**Authors:** Yuqi MENG, Zhichang YANG, Haiming FENG, Haitian LI, Bin LI

**Affiliations:** 730030 兰州，兰州大学第二医院（第二临床医学院）胸外科，甘肃省环境肿瘤学重点实验室; Department of Thoracic Surgery, The Second Hospital & Clinical Medical School, Lanzhou University; Gansu Province Key Laboratory of Environmental Oncology, Lanzhou 730030, China

**Keywords:** PYCR1, 肿瘤代谢, 信号通路, 免疫调控, 治疗靶点, 脯氨酸合成, Pyrroline-5-carboxylate reductase 1, Tumor metabolism, Signaling pathway, Immunoregulation, Therapeutic target, Proline synthesis

## Abstract

吡咯啉-5-羧酸还原酶1（pyrroline-5-carboxylate reductase 1, PYCR1）是脯氨酸生物合成途径中的关键酶，近年来在肿瘤研究领域受到广泛关注。研究表明PYCR1在多种恶性肿瘤中异常表达，通过参与肿瘤细胞代谢重编程、调控关键信号通路、影响肿瘤微环境及免疫逃逸和介导化疗耐药等机制，在肿瘤发生发展过程中发挥重要作用。本文系统综述了PYCR1的生物学结构、生物学功能、在多种肿瘤中的表达特征及其分子机制，重点探讨了其促进不同肿瘤发生发展的作用机制，以及介导化疗耐药的机制；同时还分析了PYCR1作为潜在治疗靶点的研究进展和临床应用前景，为开发新型抗肿瘤策略提供理论依据和研究方向。

## Abstract (en)

Pyrroline-5-carboxylate reductase 1 (PYCR1) is a key enzyme in the proline biosynthesis pathway and has garnered widespread attention in the field of tumor research in recent years. Studies have shown that PYCR1 is abnormally expressed in a variety of malignant tumors. It plays a significant role in tumorigenesis and development by participating in metabolic reprogramming of tumor cells, regulating key signaling pathways, influencing the tumor microenvironment, and mediating immune evasion, among other mechanisms. This article systematically reviews the biological structure and functions of PYCR1, its expression characteristics in various tumors, and the underlying molecular mechanisms. The focus is on exploring its role in promoting the occurrence and development of different tumors, as well as its involvement in mediating chemotherapy resistance. Additionally, the research progress and clinical application prospects of PYCR1 as a potential therapeutic target are analyzed, providing a theoretical basis and research directions for the development of novel anti-tumor strategies.

脯氨酸代谢重编程是肿瘤细胞适应快速增殖需求的关键特征之一。作为脯氨酸合成途径的终末酶，Δ1-吡咯啉-5-羧酸还原酶1（pyrroline-5-carboxylate reductase 1, PYCR1）催化Δ1-吡咯啉-5-羧酸（pyrroline-5-carboxylate, P5C）还原为脯氨酸，在维持细胞氧化还原稳态、能量代谢以及大分子合成中扮演核心角色^[1]^。近年来，随着代谢组学和转录组学技术的广泛应用，研究发现PYCR1在多种癌症组织中表达显著上调。PYCR1的高表达与更晚的临床分期、淋巴结转移及远处转移显著关联。此外，多项研究^[2-9]^均表明PYCR1高表达是患者预后不良的标志。

肿瘤缺氧环境下PYCR1的表达受到多种表观遗传机制的调控，同时PYCR1通过调控肿瘤细胞的关键信号通路、代谢重编程、肿瘤微环境等生物学行为，驱动癌症进展（图1）。在肿瘤中，PYCR1受到基因拷贝数变异、启动子区域调控、组蛋白修饰以及mRNA等因素的调控最终导致其过表达。PYCR1表达上调，利用NAD(P)H将底物P5C还原合成脯氨酸。这一过程消耗还原型辅酶，有助于维持细胞内的氧化还原平衡，从而支持肿瘤细胞在氧限制环境下的持续增殖和存活^[10]^。PYCR1可以激活以下通路促进肿瘤生长转移：（1）通过激活丝裂原激活蛋白激酶（mitogen-activated protein kinase, MAPK）/（extracellular signal-regulated kinase, ERK）/信号转导子与转录激活子3（signal transducer and activator of transcription 3, STAT3）信号通路促进细胞增殖并抑制凋亡^[11]^；（2）通过激活磷脂酰肌醇3-激酶（phosphatidylinositol 3-kinase, PI3K）/蛋白激酶B（protein kinase B, Akt）/哺乳动物雷帕霉素靶蛋白（mammalian target of rapamycin, mTOR）等经典促生存信号通路来驱动肿瘤生长^[12,13]^；（3）通过调控上皮-间质转化（epithelial-mesenchymal transition, EMT）相关蛋白的表达^[12]^和细胞外基质（extracellular matrix, ECM）的合成与重塑^[14]^，增强肿瘤细胞的迁移和侵袭能力。PYCR1的高表达与肿瘤免疫微环境也密切关联。PYCR1不仅可以通过调节谷氨酰胺代谢构建免疫抑制微环境^[15]^，PYCR1高表达还可以激活肿瘤细胞自噬，从而削弱CD8^+^ T细胞的抗肿瘤免疫反应^[16]^，而且PYCR1与CD8^+^ T细胞耗竭表型有关^[17]^。同时，PYCR1在肿瘤耐药性产生中也扮演了关键角色。PYCR1的高表达与多种化疗药物耐药和肿瘤复发有关^[9,18-20]^。这些研究揭示了PYCR1作为克服肿瘤耐药性潜在靶点的重要价值。

**图1:**
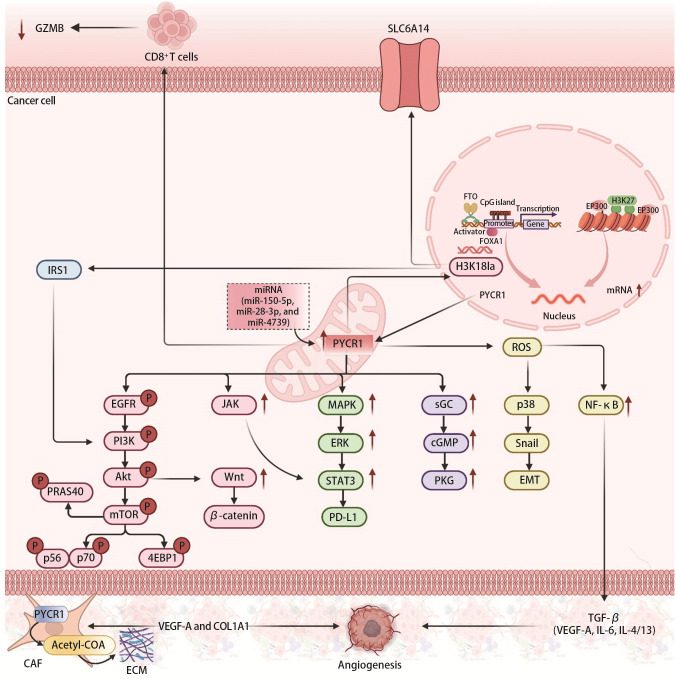
肿瘤缺氧环境下，PYCR1的表达受到多种表观遗传机制的调控，同时PYCR1通过多种途径影响肿瘤的发生发展。在肿瘤中PYCR1受到基因拷贝数变异、启动子区域调控、组蛋白修饰以及mRNA等因素的调控最终导致其过表达。PYCR1可以激活EGFR/PI3K/Akt/mTOR、Wnt/β-catenin、MAPK/ERK/STAT3、sGMP/PKG等通路促进肿瘤生长转移。PYCR1还通过调控EMT相关蛋白的表达和ECM的合成与重塑，增强肿瘤细胞的迁移和侵袭能力。PYCR1的高表达与肿瘤免疫微环境也密切关联。

PYCR1作为一个连接代谢重编程、信号转导、微环境重塑和耐药性的多功能分子，在肿瘤发生发展中发挥重要作用。深入阐明PYCR1在不同肿瘤背景下的具体作用机制，并开发其特异性抑制剂，对于实现更精准的肿瘤诊断和更有效的治疗具有重要的理论和临床意义。

## 1 PYCR1的生物学结构、功能及表达特征

### 1.1 PYCR1的生物学结构

Meng等^[21]^最早在2006年发表了关于PYCR1的生物学结构，但由于当时的晶体结构分辨率低（3.1 Å），该研究虽然提供了有关PYCR1折叠的最初信息，但配体的电子密度质量不足以定位活性位点。2017年Christensen等^[22]^发布了PYCR1的高分辨率结构（1.85-1.90 Å），该结构明确了PYCR1的活性位点、辅因子和底物。目前，在蛋白质结构数据库（Protein Data Bank, PDB）中有43个人类PYCR1的晶体结构。

近年来，随着PYCR1在肿瘤中研究的深入，关于PYCR1的结构生物学研究取得了重要进展，这些研究主要聚焦于其活性位点特征、别构调控位点的发现以及基于结构的抑制剂设计。近期发表的一项研究^[23]^通过X射线晶体学筛选片段化合物，在PYCR1的寡聚体界面发现了3个相邻的远程结合位点，这些位点距离NADH约7 Å，距离底物L-P5C 10-14 Å，共同定义了一个长达33 Å的配体结合热点沟槽。该研究证实，结合在这些别构位点的化合物能以混合型抑制机制抑制PYCR1活性，且它们不仅能与游离酶结合，也能与酶-底物-辅酶三元复合物结合。这些结构生物学进展为深入理解PYCR1的催化与调控机制，以及开发针对癌症等疾病的新型治疗策略提供了关键信息。

### 1.2 PYCR的3种不同亚型

人类基因组编码3种PYCR同工型，分别为PYCR1、PYCR2和PYCR3（亦称PYCRL），它们在亚细胞定位、肿瘤表达模式以及催化活性与底物特异性方面存在差异。在亚细胞定位上，PYCR1和PYCR2均定位于线粒体，而PYCR3则定位于细胞质^[24]^。

PYCR1和PYCR2在多种癌症中表达上调并与不良预后有关^[2,3,17,25-31]^，而PYCR3在多种肿瘤中高表达，但是在肾嫌色细胞癌、肾透明细胞癌和甲状腺癌中表达下调^[32]^。

3种同工酶均催化P5C还原为脯氨酸，但底物偏好不同。PYCR1和PYCR2都催化脯氨酸生物合成的最后一步反应，即将P5C还原为脯氨酸。两者的主要区别在于辅酶偏好：PYCR1优先利用NADH，而PYCR2则更倾向于利用NADPH^[24,33]^。PYCR3的催化途径则完全不同。它主要在细胞质中通过“鸟氨酸途径”合成脯氨酸，该途径以鸟氨酸为起始底物，最终也生成脯氨酸。这使得PYCR3能够独立于线粒体，在必要时为细胞提供脯氨酸补充^[24]^。

### 1.3 PYCR1的酶学功能及脯氨酸代谢

PYCR1是脯氨酸生物合成途径中的关键终末酶，它催化P5C在NAD(P)H的参与下还原为脯氨酸^[34]^。这一反应是脯氨酸合成的最后一步，因此PYCR1是调控细胞内脯氨酸水平的核心分子^[35]^。脯氨酸作为一种非必需氨基酸，其代谢在肿瘤细胞中扮演着至关重要的角色。它不仅是蛋白质合成的基本原料，还广泛参与细胞抗氧化、能量代谢以及ECM的重塑过程^[35]^。

PYCR1主要定位于线粒体，这一亚细胞定位与其功能密切关联^[36]^。PYCR1在线粒体中的活性不仅关乎脯氨酸的合成，还通过脯氨酸-P5C循环参与调控细胞内的氧化还原稳态。具体而言，PYCR1催化的反应消耗NAD(P)H，其产物脯氨酸又可被脯氨酸脱氢酶（proline dehydrogenase, PRODH）氧化为P5C并产生NAD(P)H或活性氧（reactive oxygen species, ROS），这一循环构成了连接细胞质与线粒体氧化还原状态的重要桥梁^[36]^。

### 1.4 PYCR1在正常组织及肿瘤组织中的表达差异

多组学数据及免疫组化分析^[3,7,27,37,38]^一致表明，PYCR1在多种肿瘤组织中表达显著上调。而且，PYCR1的高表达与肿瘤的恶性程度、临床分期及患者的不良预后密切关联^[2,3,17,27-31]^，提示其作为潜在的诊断和预后生物标志物的价值。

### 1.5 PYCR1表达调控机制

PYCR1的表达受到多种表观遗传机制的调控，包括基因拷贝数变异、启动子区域调控、组蛋白修饰以及mRNA调控等。例如在胃癌中，乙酰转移酶p300通过诱导组蛋白H3第27位赖氨酸的乙酰化（H3K27 ac）修饰促进PYCR1的转录激活，而且PYCR1启动子区域的CpG岛低甲基化与其转录水平升高显著关联^[39]^。在肺癌中FOXA1通过结合PYCR1的启动子区域，显著增强其转录活性^[16]^。在膀胱癌中去泛素化酶USP18通过稳定m^6^A去甲基化酶FTO的蛋白水平，增强了FTO的活性^[40]^。活化的FTO能够降低PYCR1 mRNA上的m^6^A甲基化水平，从而稳定PYCR1的转录本，促进其表达，最终驱动膀胱癌的发生与发展^[40,41]^。多个microRNA可以通过结合PYCR1 mRNA的3’非翻译区，促进其降解，从而抑制PYCR1的表达，例如miR-150-5p能够抑制鼻咽癌中PYCR1的表达^[42,43]^，miR-328-3p^[44]^和miR-4739^[45]^可以抑制肺癌中PYCR1的表达。

## 2 PYCR1在肿瘤发生发展中的作用机制

PYCR1作为脯氨酸合成的关键代谢酶，通过肿瘤代谢重编程、重塑免疫微环境和维持干细胞特性等机制在多种肿瘤中驱动肿瘤进展。

### 2.1 非小细胞肺癌（non-small cell lung cancer, NSCLC）

PYCR1作为脯氨酸生物合成途径中的关键酶，在NSCLC的发生发展中扮演着多重关键角色，其作用机制复杂，涉及多个信号通路和细胞过程。PYCR1可以通过调控细胞周期蛋白Cyclin D1以及线粒体凋亡通路相关蛋白Bcl-2、Bcl-xL的表达来影响细胞增殖与凋亡^[46]^。PYCR1通过调控E-钙黏蛋白表达上调以及波形蛋白、N-钙黏蛋白和Snail1的表达来调节EMT，最终促进NSCLC的侵袭与转移^[47]^。

在NSCLC中PYCR1能够与表皮生长因子受体（epidermal growth factor receptor, EGFR）和去泛素化酶USP11形成复合物，从而增强EGFR的去泛素化作用，稳定EGFR蛋白，进而持续激活下游的Akt等促生存信号^[48]^。此外，PYCR1还能与Toll样受体（Toll like receptor, TLR）信号通路的下游关键分子（如TRAF6、TAK1）相互作用，促进其泛素化和核因子κB（nuclear factor kappa-B, NF-κB）的持续活化，NF-κB的激活会驱动一系列促炎细胞因子和促生存基因的表达，为NSCLC创造一个有利于增殖、迁移和抵抗凋亡的微环境^[48]^。

在代谢重编程方面，PYCR1在NSCLC中通过调控PRODH依赖的谷氨酰胺代谢，显著激活JAK/STAT3信号通路^[8,49]^，STAT3作为转录因子，可直接结合程序性死亡配体1（programmed death ligand 1, PD-L1）基因启动子区域，上调PD-L1的表达^[49]^。免疫组化分析显示，PYCR1过表达的肿瘤组织中PD-L1表达水平显著升高，同时伴随T细胞浸润减少，提示PYCR1通过这一机制促进肿瘤免疫逃逸^[49]^。

在肿瘤免疫调节方面，PYCR1的上调会激活肺癌细胞的自噬，从而削弱CD8^+ ^T细胞的抗肿瘤免疫反应^[16]^。此外，PYCR1的高表达还能通过影响死亡受体（death receptors, DRs）在细胞膜上的重新分布，来诱导肺癌细胞对肿瘤坏死因子相关凋亡诱导配体（tumor necrosis factor-related apoptosis-inducing ligand, TRAIL）产生抵抗^[20]^。

### 2.2 乳腺癌

PYCR1在乳腺癌中表达上调并驱动肿瘤进展、干细胞特性、治疗抵抗及转移，其作用机制是多层次且复杂的。

PYCR1通过驱动脯氨酸代谢重编程，在维持乳腺癌干细胞样细胞特性中扮演核心角色。研究^[50]^表明，PYCR1合成的脯氨酸能够激活cGMP/PKG信号通路，从而增强癌症干细胞表型。重要的是，心理压力可通过该通路诱导癌症干细胞样表型和肿瘤发生，而敲低*PYCR1*可显著逆转压力诱导的脯氨酸合成、cGMP/PKG信号激活和肿瘤进展关键作用。

PYCR1不仅作用于癌细胞自身，还深刻影响肿瘤微环境。在乳腺癌的癌症相关成纤维细胞（cancer-associated fibroblasts, CAFs）中，PYCR1介导的脯氨酸合成对于产生富含胶原的促肿瘤ECM至关重要^[14]^。PYCR1在乳腺癌微环境和CAFs中均高表达，敲低CAFs中*PYCR1*可显著减少肿瘤胶原沉积，抑制肿瘤生长和转移^[14]^。表观遗传学研究^[14]^发现，乙酰辅酶A是CAFs中胶原产生的主要代谢调节因子之一，也是关键的表观遗传调节因子，CAFs中丙酮酸脱氢酶衍生的乙酰辅酶A水平升高可上调PYCR1介导的脯氨酸合成通路，这种代谢重编程为肿瘤提供了富含胶原的促癌微环境。

### 2.3 膀胱癌

PYCR1在膀胱癌中通过调控多种信号通路和细胞生物学过程，在膀胱癌的发生、发展中扮演着致癌基因的角色。在膀胱癌中PYCR1可以激活多条促癌信号通路。PYCR1可以通过调控Akt/Wnt/β-catenin^[5]^、JAK/STAT^[51]^和EGFR/PI3K/Akt^[52]^信号通路影响膀胱癌细胞的增殖、迁移和侵袭。

PYCR1也通过代谢重组途径促进膀胱癌的进展。抑制膀胱癌细胞的有氧糖酵解（表现为葡萄糖摄取、乳酸生成减少等），从而抑制肿瘤生长^[52]^。PYCR1通过增强糖酵解和乳酸产生，提高组蛋白H3K18乳酸化水平，进而上调溶质载体家族6成员14（solute carrier family 6 member 14, SLC6A14）的表达，驱动谷氨酰胺分解代谢，最终促进膀胱癌的生长和转移^[53]^。PYCR1还可以通过P5CS促进脯氨酸合成，并调节膀胱癌中的谷氨酰胺利用和代谢重编程，发挥促进肿瘤的作用^[54]^。

PYCR1还通过线粒体自噬调控膀胱癌进展。长链非编码RNA（long non-coding RNA, lncRNA）-RP11-498C9.13通过结合并稳定PYCR1 mRNA，增强其表达，进而促进ROS生成并诱导线粒体自噬，最终促进肿瘤进展^[55]^。PYCR1还通过PINK1/Parkin通路促进线粒体自噬，这一过程维持了膀胱癌干细胞的干性特征^[56]^。

### 2.4 肾细胞癌

PYCR1在肾细胞癌中发挥关键的致癌作用，其作用机制涉及代谢重编程、信号通路激活、氧化应激和免疫微环境调控等多个方面。在肾透明细胞癌^[57]^和肾乳头状细胞癌^[58]^中PYCR1均可以通过激活PI3K/Akt/mTOR信号轴促进肿瘤进展。

PYCR1驱动的代谢重编程在肾细胞癌中发挥重要作用。在肾透明细胞癌中，PYCR1通过调节谷氨酰胺代谢，参与构建一个免疫抑制性的肿瘤微环境。高PYCR1表达与更高的免疫浸润评分、更多的免疫抑制细胞浸润以及更高的免疫检查点分子表达有关^[15]^。

在肾透明细胞癌的线粒体氧化应激有关的研究^[59]^中发现，PYCR1的高表达可导致细胞内ROS水平显著降低，促进癌细胞增殖和抑制凋亡，并降低癌细胞对5-氟尿嘧啶等化疗药物的敏感性；而且PYCR1与SLC25A27等线粒体转运蛋白共同构成一个可预测患者生存的分子特征^[30]^。

转移性肾细胞癌中的研究^[60]^支持PYCR1的免疫抑制作用，高表达PYCR1与CD8^+^ T细胞耗竭表型有关，表现为颗粒酶B表达降低，并且与免疫治疗联合酪氨酸激酶抑制剂的耐药性有关。

### 2.5 前列腺癌

前列腺癌干细胞中，代谢组学分析^[61]^发现尿素循环异常和脯氨酸代谢亢进是干性维持的特征性改变。PYCR1在前列腺癌干细胞中高表达，其催化的脯氨酸合成可通过激活JAK2/STAT3信号通路促进干性特征。研究^[61]^证实PYCR1抑制剂可显著降低肿瘤球形成能力和移植瘤生长，同时伴随干性标志物表达下调；此外，前列腺癌中还发现脂肪酸代谢重编程特征，包括肉碱和游离脂肪酸水平升高，以及鞘磷脂减少/甘油三酯增加等脂质代谢异常。

### 2.6 肝细胞癌

PYCR1通过驱动代谢重编程并激活多种致癌信号通路以及参与表观遗传调控等机制，促进肝细胞癌发生发展的复杂网络。

在肝细胞癌中PYCR1和ALDH18A1等脯氨酸合成酶在肝癌中表达上调，而脯氨酸分解酶如PRODH则下调^[62]^。在缺氧条件下PYCR1促进肝细胞癌中的脯氨酸合成，通过重编程肝细胞癌的氨基酸代谢，最终通过MAPK/ERK/STAT3 信号通路促进肿瘤进展^[11]^。

PYCR1的高表达能显著增强细胞的糖酵解水平，导致乳酸产量增加。这些过量的乳酸可作为前体物质，催化H3K18发生乳酸化修饰。PYCR1介导的H3K18乳酸化修饰会特异性富集在胰岛素受体底物1（insulin receptor substrate-1, *IRS1*）基因的启动子区域，进而调控IRS1的转录表达^[13]^。IRS1是PI3K/Akt/mTOR和MAPK/ERK等关键促生长信号通路的上游调控因子，因此，PYCR1介导的H3K18修饰通过上调IRS1表达，最终促进了肝癌细胞的增殖与转移^[13]^。

### 2.7 多发性骨髓瘤（multiple myeloma, MM）

在MM中缺氧骨髓微环境诱导PYCR1过表达，然后影响PRAS40、mTOR、p70、pS6等蛋白的磷酸化水平，从而促进蛋白质合成^[63]^。

### 2.8 胶质母细胞瘤

研究^[64]^表明PYCR1在胶质瘤CAFs中高表达，并通过促进胶原蛋白合成和血管内皮生长因子（vascular endothelial growth factor, VEGF）分泌来支持肿瘤进展。在胶质母细胞瘤模型中PYCR1通过上调CAFs分泌的I型胶原蛋白α1（collagen type I alpha 1, COL1A1）和VEGF-A等因子，重塑肿瘤微环境以促进恶性进展；通过荧光激活细胞分选技术分离不同PYCR1表达水平的CAFs后，发现PYCR1高表达CAFs与C6胶质瘤细胞共培养时，能显著增强肿瘤细胞的侵袭、增殖能力以及微血管内皮细胞的血管生成能力。

### 2.9 结肠癌

PYCR1通过直接与STAT3相互作用，进而调控STAT3介导的p38 MAPK和NF-κB信号通路，从而影响细胞的增殖、耐药和EMT表型^[65]^。

PYCR1可能参与结肠癌细胞铁死亡途径，铁死亡诱导剂（Erastin）可以抑制PYCR1过表达所产生的促肿瘤效应^[19]^。在结肠癌中，PYCR1在缺氧环境下被招募到转录因子ELK4靶基因的启动子区域。其催化活性所产生的NAD+，是III类组蛋白去乙酰化酶Sirt7的必需辅因子。NAD+的存在激活了Sirt7，使其能够催化去除H3K18的乙酰化修饰。这种组蛋白去乙酰化作用导致相关基因的转录抑制，从而帮助肿瘤细胞在缺氧压力下维持生长^[66]^。

### 2.10 胰腺癌

针对胰腺癌CAFs的靶向治疗研究^[67]^显示，通过工程化外泌体递送miR-138-5p可抑制FERMT2-PYCR1复合物形成，阻断脯氨酸介导的胶原合成，从而重塑纤维化微环境并增强化疗敏感性。

### 2.11 食管鳞状细胞癌

在食管鳞状细胞癌中，PYCR1与EGFR的直接相互作用被证实是激活该通路的关键机制，通过免疫共沉淀和质谱分析发现，PYCR1-EGFR复合物的形成显著增强了PI3K/Akt/mTOR通路的活性，进而促进肿瘤细胞的迁移和侵袭能力^[68,69]^。

### 2.12 口腔鳞状细胞癌

在口腔鳞状细胞癌中，线粒体伴侣蛋白Lon的上调通过PYCR1诱导线粒体ROS的产生，而ROS依赖性地激活了p38和NF-κB信号通路^[70]^。活化的NF-κB进一步促进了转化生长因子-β、白细胞介素（interleukin, IL）-6、IL-13和VEGF-A等炎症细胞因子的产生，这些因子共同作用，驱动EMT转化、血管生成以及M2型巨噬细胞极化，从而塑造并维持了一个免疫抑制性的肿瘤微环境，最终促进癌症的进展和转移^[70]^。

## 3 PYCR1介导的化疗耐药机制

PYCR1通过调控脯氨酸代谢，在多种肿瘤中介导化疗耐药，其机制主要涉及维持细胞氧化还原平衡、支持肿瘤干细胞特性以及影响肿瘤微环境。在食管鳞状细胞中，缺氧微环境通过上调胰岛素样生长因子-1受体（insulin-like growth factor 1 receptor, IGF-1R）信号通路，进而促进c-MYC介导的PYCR1表达增强，PYCR1的激活增强了脯氨酸代谢，这对于维持细胞内的氧化还原平衡至关重要，使得肿瘤细胞能够在顺铂治疗的压力下持续增殖，从而产生耐药性^[71]^。在乳腺癌中，基于质谱的蛋白质组学分析发现，PYCR1是脯氨酸生物合成途径中的关键蛋白，其表达水平与患者对化疗的反应不良及肿瘤复发显著关联^[9]^。此外，PYCR1合成的脯氨酸能够激活cGMP/PKG信号通路，从而增强乳腺癌干细胞样特性，这种特性与肿瘤的转移、耐药和复发密切关联^[50]^。在结肠癌中PYCR1通过上调线粒体二羧酸载体蛋白SLC25A10的表达，抑制5-氟尿嘧啶诱导的脂质ROS产生和铁死亡，从而削弱该化疗药物的细胞毒性作用。使用铁死亡诱导剂Erastin可模拟PYCR1敲低的抗肿瘤效果，而铁死亡抑制剂则能逆转PYCR1敲低带来的增敏作用^[19]^。

PYCR1的高表达与肿瘤细胞对多种化疗药物的耐受性有直接关联。在胰腺癌中肿瘤微环境中的CAFs通过FERMT2-PYCR1复合物介导脯氨酸代谢，促进胶原合成，从而加剧纤维化和化疗耐药。靶向抑制该复合物（如通过工程化细胞外囊泡递送miR-138-5p）能够重塑肿瘤微环境，改善吉西他滨的灌注并增强癌细胞对化疗的敏感性^[67]^。在MM中PYCR1的高表达与患者较差的总体生存率有关，并且复发/难治性患者的PYCR1 mRNA水平显著升高。抑制PYCR1可以降低PRAS40和mTOR等蛋白的磷酸化水平，最后通过抑制PRAS40介导的蛋白质合成来显著增强其对蛋白酶体抑制剂硼替佐米的敏感性^[63]^。此外，抑制骨髓基质细胞中的PYCR1，会减少其分泌激活素A，从而限制MM细胞的氧化磷酸化并增强硼替佐米的疗效，这揭示了肿瘤微环境中基质细胞代谢对癌细胞耐药性的间接贡献^[18]^。在肾透明细胞癌中，FOXM1通过正调控PYCR1，激活PI3K/Akt/mTOR信号轴并促进血管生成拟态形成，这与化疗耐药增强和不良预后有关^[57]^。这些研究共同表明，PYCR1是连接肿瘤代谢重编程与化疗耐药的关键节点，靶向PYCR1或其下游通路有望成为逆转耐药、改善癌症治疗效果的潜在策略。

## 4 PYCR1在临床转化中的研究

### 4.1 PYCR1作为肿瘤生物标志物的潜力

PYCR1在多种肿瘤组织（如骨肉瘤^[72]^、胰腺癌^[7]^、头颈部鳞癌^[73]^和肺腺癌^[8]^等）中均呈现高表达，并与患者的不良预后显著关联（如膀胱癌^[17,29,54]^、胃癌^[27]^、肾癌^[25,59,74-76]^、肺癌^[49,77]^和头颈部鳞癌^[28]^等），这使其具备了成为诊断和预后评估指标的巨大潜力。除了组织水平的表达，肺癌^[49]^和口腔鳞癌^[78]^患者的血清PYCR1水平也显著升高，显示出作为非侵入性肿瘤监测手段的潜力。对PYCR家族（包括PYCR1）的泛癌分析也显示，PYCRs在多种肿瘤中表达升高，并与不利的临床结局有关，特别是在肾透明细胞癌中基于PYCR1和PYCR2的预后模型具有独立的预测价值^[32]^。

### 4.2 靶向PYCR1的治疗策略研究

PYCR1作为癌症治疗靶点受到广泛关注，其抑制剂研究近期在化合物发现、作用机制及临床前应用方面取得了重要进展。

Ragin-Oh等^[79]^通过多种筛选策略发现了结构多样的PYCR1抑制剂。晶体片段筛选首次系统性地探索了PYCR1的结合口袋，鉴定出磺酰胺和磺胺酸酯等可作为底物羧酸基团等排替代的新化学片段，并发现了靠近烟酰胺结合位点的隐蔽亚口袋。通过基于结构的虚拟筛选与分子动力学模拟，天然蒽醌化合物大黄素被鉴定为PYCR1的结合剂^[80]^。此外，已报道的选择性抑制剂PYCR1-IN-1在肺癌模型中证实了其药理活性^[48]^，而Pargyline则在MM的微环境研究^[18]^中被用作PYCR1抑制剂。生物信息学分析^[81]^也预测了多个具有潜在抑制活性的小分子。近期研究^[23]^具有突破性的发现是揭示了PYCR1的变构抑制剂结合区，该位点位于活性位点远端（距离NADH约7 Å，距离底物L-P5C 10-14 Å），是一个跨越约33 Å的配体结合热点沟槽；与此前所有结合在活性位点的抑制剂不同，这些变构抑制剂以混合型抑制机制发挥作用，并且能够与酶-底物-辅酶三元复合物结合。

在治疗靶点开发方面取得了一定的进展，仍需注意以下几点：（1）PYCR1除了在多种癌症中高表达之外在部分正常组织中也有功能。因此，抑制剂的肿瘤选择性至关重要。癌细胞因其高速增殖而更依赖PYCR1支持的脯氨酸和胶原蛋白合成，但如何量化这种依赖性差异并转化为治疗窗口仍需探索。（2）虽然靶向PYCR1的小分子抑制剂（如Pargyline）在临床前研究^[63]^中已显示出增敏化疗的潜力，仍需进一步开发高选择性PYCR1抑制剂。（3）不同肿瘤类型中PYCR1的调控网络存在明显异质性，例如在结直肠癌中Wnt/β-catenin通路占主导，而在胶质瘤中则更多依赖JAK/STAT3通路激活，这种组织特异性提示需要开发个体化的治疗策略。未来的临床开发需在特定肿瘤类型背景下，基于其独特的调控机制来设计试验和选择患者。

### 4.3 PYCR1在肿瘤免疫治疗中的应用前景

PYCR1作为脯氨酸代谢通路的关键酶，近年来被发现在肿瘤免疫微环境调控中发挥重要作用。多项研究^[15,16,54,60]^表明，PYCR1通过影响肿瘤细胞的代谢重编程，直接参与免疫抑制性微环境的形成。靶向PYCR1的肿瘤免疫治疗策略可能包括3个方向：（1）开发PYCR1特异性抑制剂与免疫检查点抑制剂（immune checkpoint inhibitors, ICIs）的联合方案；（2）基于PYCR1表达水平建立预测模型，指导免疫治疗患者分层；（3）针对PYCR1下游代谢-免疫调控网络设计多靶点干预方案。值得注意的是，泛癌分析显示PYCR1在多种肿瘤中高表达且与不良预后有关，但其免疫调控机制存在组织特异性差异^[32]^。因此，未来研究需要进一步阐明PYCR1在不同肿瘤微环境中的精确作用机制，并通过前瞻性临床试验验证联合治疗策略的临床效益。随着对肿瘤代谢-免疫相互作用认识的深入，PYCR1有望成为改善肿瘤免疫治疗疗效的新靶点。

## 5 小结与展望

PYCR1作为脯氨酸代谢途径的核心调控酶，其异常表达与肿瘤发生发展的多维度关联已得到广泛证实。从分子机制层面来看，PYCR1通过整合代谢重编程与信号转导网络，构建了独特的肿瘤促进模式：一方面通过维持脯氨酸-谷氨酰胺循环维持氧化还原稳态和能量供应，另一方面通过PI3K/Akt等经典通路与表观遗传修饰的交叉对话，形成正反馈调节环。这种多靶点作用特征使其在肿瘤干细胞维持、EMT转化和免疫微环境重塑等关键生物学过程中扮演枢纽角色。

现有研究揭示了PYCR1在肿瘤的诊断和治疗中的双重价值。未来研究应重点关注3个维度：首先需解决现有抑制剂生物利用度低和血脑屏障穿透性差的技术瓶颈，通过纳米载体递送或前药改造提升药效；其次应建立PYCR1表达水平与化疗敏感性的预测模型，指导临床联合用药方案的优化；最重要的是开展多中心临床试验验证其作为“代谢检查点”的治疗价值，特别是与现有靶向治疗的协同效应。从转化医学视角看，PYCR1研究的突破不仅丰富了肿瘤代谢理论，更开辟了通过干预氨基酸代谢重塑肿瘤微环境的治疗新范式，但其临床应用仍需平衡治疗效果与代谢干预可能引发的系统性副作用。
